# Association between sociodemographic status and the T2DM-related risks in China: implication for reducing T2DM disease burden

**DOI:** 10.3389/fpubh.2023.1297203

**Published:** 2024-01-08

**Authors:** Xin Huang, Yinhui He, Haiyan Xu, Yuyan Shen, Xiaowen Pan, Junyun Wu, Kai Chen

**Affiliations:** ^1^Department of Endocrinology and Metabolism, Lishui Municipal Central Hospital, Lishui, China; ^2^Department of Endocrinology and Metabolism, The Second Affiliated Hospital of ZheJiang University School of Medicine, Zhejiang, China

**Keywords:** type 2 diabetes mellitus, China, disability-adjusted life years, high body mass index, ambient particulate matter pollution, sociodemographic index

## Abstract

**Objective:**

Analyzing the association between sociodemographic status and the type 2 diabetes mellitus (T2DM)-related risks in China to reduce the disease burden of T2DM.

**Methods:**

We downloaded data from the Global Burden of Disease Study 2019 to estimate the disease burden of T2DM in China. Secondary analyses were performed by year, age, gender, summary exposure value (SEV), and sociodemographic index (SDI).

**Results:**

In China, it is estimated that 3.74 (3.44–4.10) million incidence, 90.0 (82.3–98.5) million prevalence, 168.4 (143.2–194.0) thousand deaths, and 9.6 (7.6–11.9) million DALYs occurred in 2019, showing an increase of 96.8, 156.7, 162.8, and 145.4% compared to 1990. An inverse U-shaped curve was observed for the correlations between T2DM-related burden and SDI. A heavier burden was found in males. The top four risk factors were high body mass index (HBMI), dietary risks, air pollution and tobacco. HBMI, as the key risk, accounted for half of the disease burden of T2DM in China. Lower degree of SEV and higher level of attributable T2DM-related burden could be found in main risks, meaning their critical role of them in the development and progression of T2DM. An inverse U-shaped curve could be found in the association between age-standardized incidence, mortality, DALYs rate, and SDI.

**Conclusion:**

The disease burden of T2DM has rapidly increased in China. Gender disparities, different age distributions and inconsistent socioeconomic levels all played an important role in it. The key risk was HBMI. With the improvement of socioeconomic level, the main risk factors for T2DM have changed from environmental factors to lifestyle factors. Targeted control and preventative strategies to address adjustable risk factors could put an end to this soaring burden.

## Introduction

As revealed in the International Diabetes Foundation’s Diabetes Atlas 2021(IDF 2021), 10.5% of the global population lives with diabetes, and over three in four adults with diabetes live in low- and middle-income countries. This number was projected to rise to 643 million by 2030 and 783 million by 2045 (1). Reported by The World Health Organization (WHO), diabetes was not only one of the four main noncommunicable diseases but also one of the top 10 leading causes of death (with an average of one person dying of diabetes every 5 s) (2). Vascular complications of both the macrovascular system (cardiovascular diseases) and microvascular system (diabetic kidney diseases, diabetic retinopathy, and neuropathy) have led to increasingly heavier mortality, disability (blindness, kidney failure, heart failure, and amputation), and an overall decreased quality of life, carrying an enormous financial burden (at least 966 billion dollars in 2021 increased 316% over the last 15 years).

Type 2 diabetes mellitus (T2DM) is affected by many factors, such as genetics, environment, and behavioral habits. The main risk factors include obesity, smoking, poor eating patterns, secondhand smoke exposure, and insufficient physical activity. High body mass index (HBMI) is the leading risk factor for many diseases. In 2017, 16.7% of cardiovascular disease cases, 28.6% of T2DM and its chronic kidney disease cases, and 4.8% of cancer cases could be attributed to HBMI (3). Air pollution is also an important issue. Recent studies have shown that every 10-μg/cubic meter increase in PM2.5 will lead to a 36% increase in diabetes-related mortality (4). In addition, a reasonable dietary structure is also particularly important, including whole grains, nuts, and other dietary supplements. The intake of fiber, fresh vegetables and fruits, and healthy dietary patterns such as the Mediterranean diet did help to control blood sugar, blood pressure, and blood lipids, while the intake of high-sugar drinks, high-energy foods, and processed meat products have the opposite effect (5). According to the Diabetes Prevention Study in the United States and the Netherlands, after 10 years of intensive lifestyle intervention, participants who lost >5% of their body weight and those who lost 3–5% of their body weight, respectively, had a 64 and 40% lower risk of developing T2DM during 6 years of follow-up (6). Reasonable control of these modifiable risk factors, such as metabolism, environment, and dietary behavior, can significantly reduce the disease burden of T2DM.

In addition, sociodemographic factors such as population aging, racial differences, urbanization, and socioeconomic factors such as education level, income level, and medical and health resources are also important in affecting the disease burden of T2DM. Benefiting from the economic development and improvement in healthcare, in many developed countries, including Scotland, Canada, the UK, Denmark, Sweden, Australia, and the United States, T2DM-related mortality has declined steadily (7). While in developing countries, societal shifts, lifestyle changes, limited resources, and inaccessibility to medications all contribute to the ongoing and increasing trends of T2DM and high-T2DM-related disability (6, 7). As predicted by an epidemiological study, during 2010–2030, the number of adults with diabetes mellitus in developing countries would increase by 69% (3-fold in developed countries) (8). One study showed that all-age prevalence of diabetes nearly doubled to 6.6% from 1990 to 2017, and all-age mortality rates of diabetes and diabetes-related CKD increased by 63.5 and 33.3%, respectively. Patients aged 15–49 years increased more rapidly than in any other age group, and a heavier burden was found in males.

High BMI, air pollution, a diet low in whole grains, and smoking were the most important risk factors in 2017 (9). A continuing upward trend in HBMI has shifted the onset of diabetes to the younger population, particularly women under the age of 50, especially those in developed countries, contributing to the increase in the incidence of diabetes (10). Moreover, most grains consumed are refined, such as refined wheat flour and white rice, which are major contributors to the dietary glycemic load associated with an increased risk of diabetes (11). Over time, higher total energy intake and a higher proportion of energy intake from carbohydrates have increased in popularity (12). Other changes in dietary patterns include eating away from home and increased consumption of sweetened beverages and low-nutrient-dense foods, which also contributed to the rise in T2DM through their direct effects on body weight (13). In previous studies, participants who consumed more sugar-sweetened beverages had higher incidence rates of hypertension, dyslipidemia, and T2DM (14, 15). A recent study found that a higher total carbohydrate intake was associated with increased mortality. It is well known that both aerobic and resistance forms of exercise improve glycemic control in patients with T2DM and are a key component of diabetes prevention strategies (16–18). Moderate to high levels of physical activity and cardio-respiratory fitness have also been associated with reduced mortality in individuals with diabetes (19).

China is the largest developing country; the number of people suffering from diabetes has reached 140 million in 2021 (1/4 of the world’s total diabetes), of which more than 90% are T2DM. Moreover, many people are still undiagnosed. Societies shifts, population aging, lifestyle changes (especially the epidemic of obesity, changes in eating patterns, and low physical activity), urbanization, limited resources, industrialization, inaccessibility to medications, and inconvenient treatments, these all contributed to the increasing burden of T2DM. Given the current situation in China, it is crucial to explore the trends of T2DM-related burden and its attributable risks to provide guidance for policymakers to adjust health-promoting and disease-preventative strategies. Thus, in this study, we aimed to estimate T2DM-related burden by incidence, prevalence, deaths and DALYs attributable to various modifiable risk factors from 1990 to 2019 in China, based on The Global Burden of Disease Study 2019 (GBD 2019).

## Methods

### Data source

Data were extracted from the GBD 2019^1^ (20) included: (1) gender-specific data on incidence, prevalence, deaths and DALYs as all-ages numbers and age-standardized rates by year in China; (2) gender-specific prevalence, deaths and DALYs as all-ages numbers and age-standardized rates by age groups in 2019 in China; (3) gender-specific deaths and DALYs attributable to the most-detailed risk factors as absolute numbers and age-standardized rates in 1990, 2005, and 2019 in China; and (4) SDI data and gender-specific SEV attributable to the most-detailed risk factors by year in China. The data were accessible to the public, so informed consent and ethics approval were not required for this study. There is no risk of disclosing the identity of individuals.

### Risk factors

Risk factors are defined as an attribute, behavior, exposure, or other factors which are causally associated with an increased (or decreased) probability of a disease or injury. If the probability decreases, the risk is a protective factor. The GBD 2019 classified risk factors of diabetes into environmental/occupational risks, behavioral risks, and metabolic risks, specifically including 17 of the most-detailed risk factors: high-fasting plasma glucose, HBMI; ambient particulate matter pollution (APMP), household air pollution from solid fuels, exposure to high temperatures or low temperatures; low physical activity, a diet low in whole grains, nuts, seeds, fiber, or fruit, a diet high in red meat, processed meat, or sweetened beverages, alcohol use, smoking, and secondhand smoke (21). All relevant risk factor definitions, classification criteria and attributable disease burden codebooks can be found in my Supplementary material.

### The sociodemographic index

As a measure of sociodemographic development, SDI was a composite measure based on the total fertility rate in people younger than 25 years, educational attainment in people older than 15 years and income *per capita*. Calculated by GBD 2019, the SDI ranges from 0 to 1, and a higher value indicates a higher socioeconomic status. The SDIs of the GBD 2019 countries were divided into five categories as follows: high SDI (>0.81), high-to-middle SDI (0.70–0.81), middle SDI (0.61–0.70), low-to-middle SDI (0.46–0.61) and low SDI (<0.46) (21).

### Summary exposure value

A measure of a population’s exposure to a risk factor that considers the extent of exposure by risk level and the severity of that risk’s contribution to disease burden. SEV takes the value zero when no excess risk for a population exists and the value one when the total population is at the highest level of risk; we report SEV on a scale from 0 to 100% to emphasize that it is risk-weighted prevalence (21).

### DALYs and annual growth rate

The DALYs are defined as the sum of years lost due to premature death (YLLs) and years lived with disability (YLDs). DALYs are also defined as years of healthy life lost. YLLs are the multiplication of deaths and a standard life expectancy at the age of death. The standard life expectancy is derived from a life table that contains the lowest observed mortality rate at each age that has been observed in any population greater than 5 million. YLDs are defined as years lived with any short-term or long-term health loss weighted for severity by the disability weights (22, 23). The rate of annual growth was measured as the average annual change in type 2 diabetes burden from 1990 to 2019.


$$\text{Annual}\;\text{growth}=\frac{\text{The}\;\text{value}\;\text{of}\;2019-\text{The}\;\text{value}\;\text{of}\;1990}{\text{The}\;\text{value}\;\text{of}\;1990\times 29}$$

### Statistical analysis

Data are performed as absolute values with a 95% uncertainty interval (UI). Age-standardized rates of deaths and DALYs are expressed as the number per 100,000 population. All figures and statistical analyses were conducted using GraphPad Prism (version 9.0) and Echarts open-source system. A *p*-value of less than 0.05 is considered a significant difference.

## Results

### The trends of the disease burden of T2DM in China by time and gender

**Figure 1:**
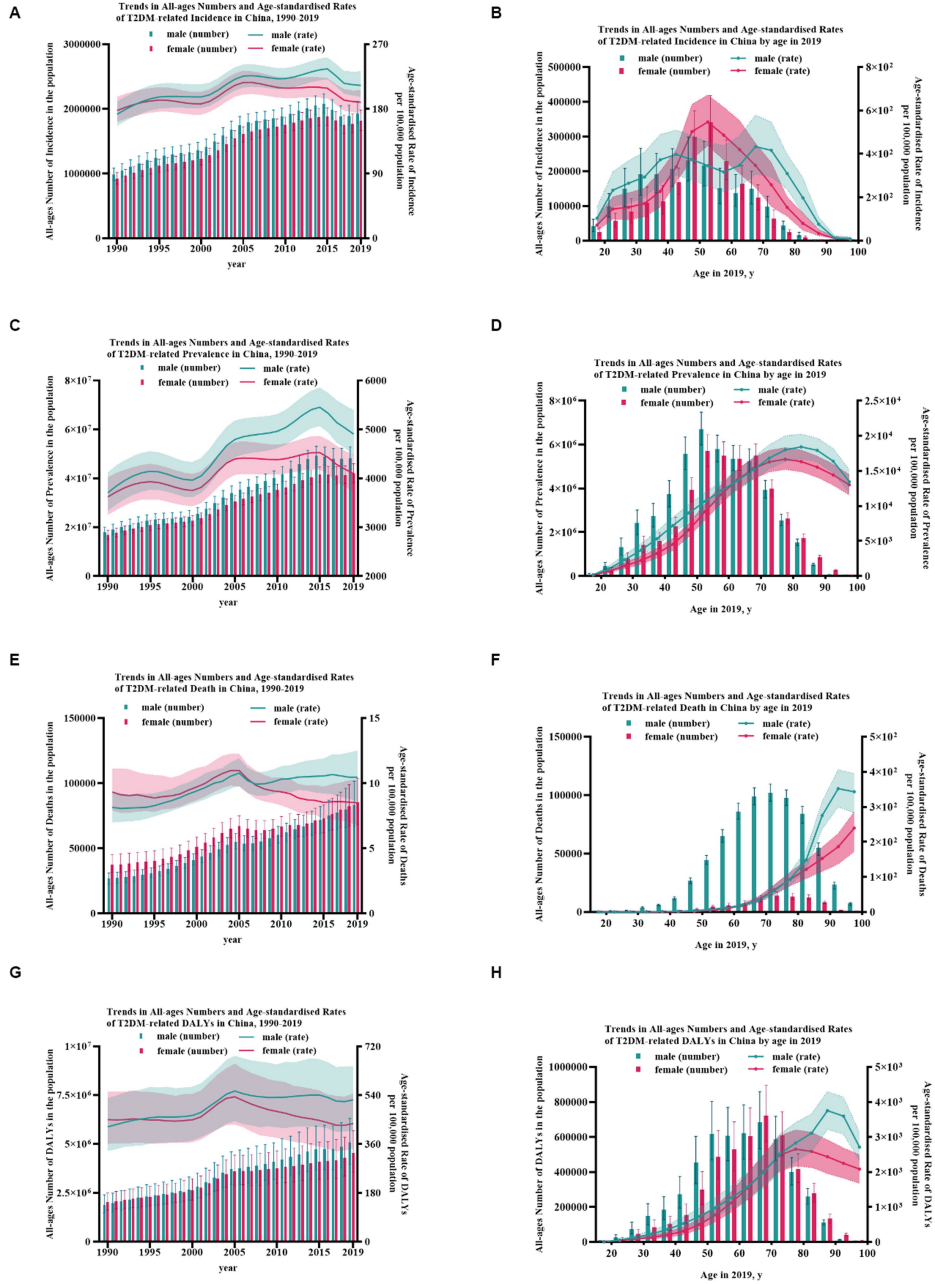
The burden of T2DM from 1990 to 2019 in China. **(A)** Trends in all-ages numbers and age-standardized rates of T2DM-related incidence in China, 1990–2019; **(B)** trends in all-ages numbers and age-standardized rates of T2DM-related incidence in China by age in 2019; **(C)** trends in all-ages numbers and age-standardized rates of T2DM-related prevalence in China, 1990–2019; **(D)** trends in all-ages numbers and age-standardized rates of T2DM-related prevalence in China by age in 2019; **(E)** trends in all-ages numbers and age-standardized rates of T2DM-related death in China, 1990–2019; **(F)** trends in all-ages numbers and age-standardized rates of T2DM-related death in China by age in 2019; **(G)** trends in all-ages numbers and age-standardized rates of T2DM-related DALYs in China, 1990–2019; and **(H)** trends in all-ages numbers and age-standardized rates of T2DM-related DALYs in China by age in 2019. Error bars indicate the 95% uncertainty interval (UI) for numbers. Shading indicates the 95% UI for rates.

In China, it is estimated that 90.0 (95% UI: 82.3–98.5) million cases, 3.74 (3.44–4.10) million new cases, 168.4 (143.2–194.0) thousand deaths, and 9.6 (7.6–11.9) million DALYs attributed to T2DM occurred in 2019, showing a significant increase of 96.8, 156.7, 162.8, and 145.4% compared to 1990, respectively. However, a slight uptrend was found in the age-standardized rate of T2DM-related burden. The age-standardized rate of prevalence and incidence rose by 22.5% to 4496.4 (95% UI: 4097.9–4928.8) per 100,000 population and 15.4% to 201.1 (185.7–219.2) per 100,000 population, peaking in 2015. Similarly, the age-standardized mortality and DALYs rate rose slightly by 7.0% to 9.2 (7.9–10.5) per 100,000 population and 9.3% to 476.5 (376.4–589.5) per 100,000 population, peaking in 2005. Interestingly, the death number of T2DM was found to be heavier in females, while heavier in males for the others. Estimated by age-standardized rate of prevalence, incidence and DALYs rate, a heavier burden was found in males. As for mortality rate, the curves of the two genders showed an intersection in the year 2007, after which the mortality rate among males exceeded that of females (Figure 1).

### The trends of the disease burden of T2DM in China by age and gender

The number of T2DM cases peaked at age 50–54 without gender difference, with males at 6.70 (95% UI: 5.98–7.48) billion and females at 5.73 (4.99–6.45) billion. As for the number of deaths, both genders peaked at age 70–74, with males at 14.1 (11.2–17.4) thousand and females at 15.5 (UI: 12.3–19.1) thousand. Similarly, the number of DALYs, without gender difference, peaked at age 65–69, with males at 685.6 (532.1–859.2) thousand and females at 721.5 (575.2–896.2) thousand. The curves of males and females showed an intersection at age nearly 62, before which the number of prevalence, incidence, deaths, and DALYs among females was lower than that of the males. This may partly benefit from the protective effect of estrogen and female’s long life. The crude of morbidity increased with age gradually, in males peaking at age 80–84 at 18.4 (16.7–20.2) thousand per 100,000 population but age 75–79 at 16.7 (15.2–18.3) thousand per 100,000 population in females. As for mortality, it rose substantially in females with age, while in males, it peaked at 90–94 at 351.9 (299.5–406.0) per 100,000 population. The peak rate of DALYs appeared at age 75–79 at 2652.6 (2165.1–3206.1) per 100,000 population in females and age 85–89 in males at 3751.0 (3215.4–4299.0) per 100,000 population. A heavier burden was found in males for nearly all age groups (*p* < 0.05). Age groups with the largest gender differences were 40–44 in morbidity, 90–94 in mortality, and 85–89 in DALYs rate (Figure 1; Supplementary Figure S2).

### The trends of the disease burden of T2DM attributable risk factors by time in China

In 2019 in China, the leading three risk factors were HBMI, ambient particulate matter pollution (APMP) and smoking. Dietary factors were the ones with the greatest change in ranking. Specifically, a diet high in red meat, a diet low in whole grains and diet high in processed meat achieved a huge upward shift in ranking, while a diet low in fruits, a diet high in sugar-sweetened beverages and diet low in fiber have declined a lot. In these 30 years, household air pollution from solid fuels has achieved five downward shifts in ranking overall. The key contributing factor was HBMI, accounting for 28.2% of the T2DM-related deaths at 47.5 (22.5–76.6) thousand and 38.9% of the T2DM-related DALYs at 3.74 (1.91–5.90) million.

HBMI, as the main risk, the age-standardized death rate had increased from 1.2 (0.3–2.6) per 100,000 population to 2.4 (1.1–3.9) per 100,000 population, doubled from 1990. It was followed by APMP (rose from 0.7 [0.3–1.2] to 1.7 [1.2–2.3] per 100,000 population) and smoking (elevated from 1.0 [0.8–1.2] to 1.1[0.8–1.4] per 100,000 population). These three risk factors collectively contributed to nearly half (57.1%) of all T2DM-related deaths rate in 2019 in China. Similarly, the age-standardized DALY rates attributable to HBMI increased from 80.2 (95% UI: 21.5–167.4) per 100,000 population to 181.5 (93.7–286.3) per 100,000 population, showing an increase of 126.3%, followed by APMP (increased from 32.6 [14.2–59.1] to 89.7 [59.6–123.4] per 100,000 population) and smoking (rose from 61.8 [47.6–79.6] to 68.4 [50.8–87.0] per 100,000 population). These factors mentioned above totally accounted for 74.62% of T2DM-related DALYs.

**Figure 2:**
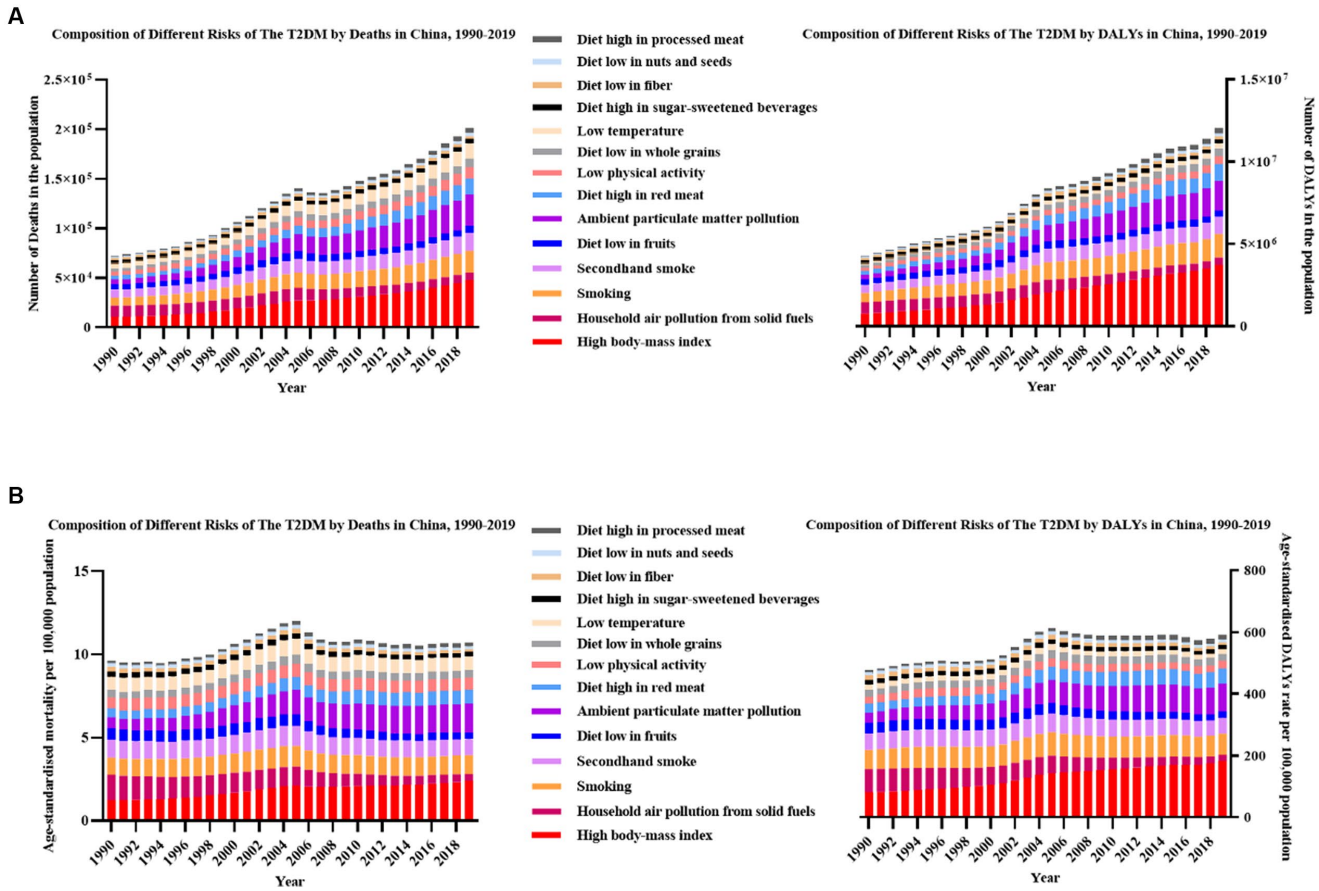
The most-detailed risk factors attributable to T2DM-related burden in China, 1990–2019. **(A)** All-ages number of deaths and DALYs; **(B)** age-standardized rate of deaths and DALYs.

**Figure 3:**
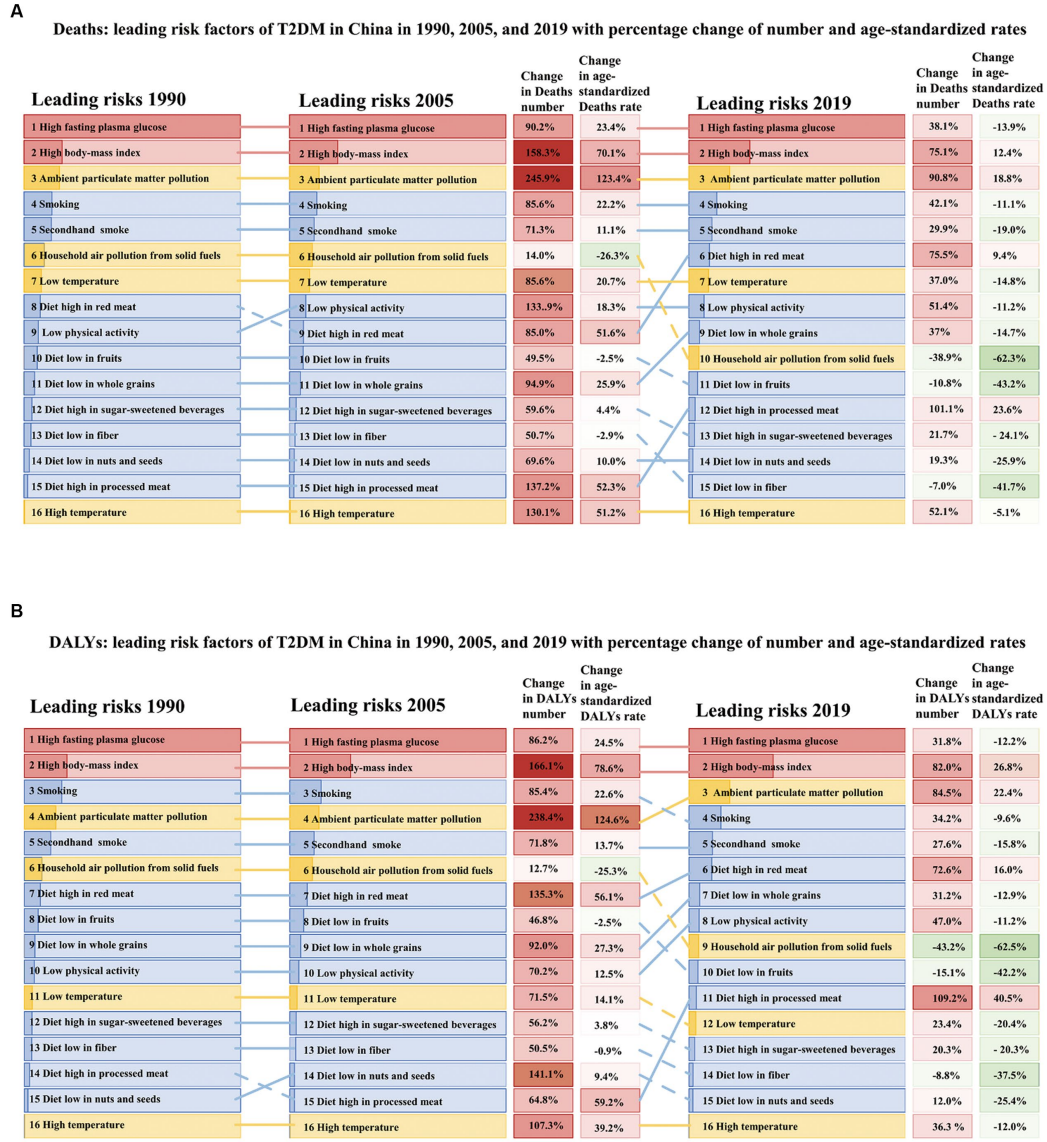
The most-detailed risk factors attributable to T2DM-related burden in China in 1990, 2005, and 2019, with percentage changes in the all-ages numbers and age-standardized rates. **(A)** Number and rate of deaths and **(B)** number and rate of DALYs. Solid lines indicate increases and dashed lines indicate decreases in rank between periods. The darker part in each column represents the proportion of corresponding causes. The red part is metabolic risk factors, the blue part is behavioral risk factors, and the yellow part is environmental risk factors. Red indicates that the rate of change is positive, while green is negative, and the depth of color indicates the degree of change.

Considering the annual growth rate of T2DM-related age-standardized rates, the top three risk factors that increased the most were APMP, HBMI, and a diet high in processed meat (rose by 5.7, 3.1, and 3.0% in deaths rate and increased by 6.0, 4.4, and 4.3% in DALYs rate, respectively). Instead, the top three risk factors that declined most were household air pollution from solid fuels, a diet low in fruit and a diet low in fiber (decreased by 2.5, 1.5, and 1.5% in deaths rate and declined by 2.5, 1.5, and 1.3% in DALYs rate, respectively). The risk factor ranking third was secondhand smoke in females, while in males, it was smoking. Estimated by DALYs attributable to tobacco, men were more affected by smoking (smoking vs. secondhand smoke 24.46% vs. 7.12%), whereas women were secondhand smoke (secondhand smoke vs. smoking 15.04% vs. 2.70%). The same phenomenon could be found in death (Figures 2; 3, Table 1).

**Table 1:** All-ages number of deaths and DALYs, the age-standardized rate of deaths and DALYs and its annual growth rate with the 95% uncertainty interval (UI; lower, upper) of T2DM in China in 1990 and 2019 by gender.

GBD 2019 risk factors in China		All-ages deaths number (×10^2^, 95% CI)			Age-standardized deaths rate (/100,000, 95% CI)		
		1990	2019	Annual growth%	1990	2019	Annual growth%
**Females**							
Lifestyle	High BMI	64.9 (19.1, 131.5)	247 (115.5, 405)	9.7%	1.5 (0.4, 3)	2.4 (1.1, 3.9)	2.0%
	Low physical activity	28.3 (13.3, 49.9)	70.5 (34, 123.4)	5.1%	0.8 (0.4, 1.3)	0.7 (0.4, 1.3)	−0.2%
Environmental pollutants	Ambient particulate matter pollution	25.1 (11, 46.5)	155.6 (103.1, 214.9)	17.9%	0.6 (0.3, 1.2)	1.6 (1, 2.1)	5.0%
	Household air pollution from solid fuels	69.7 (44.3, 106.3)	45.7 (22.6, 78.9)	−1.2%	1.7 (1.1, 2.6)	0.5 (0.2, 0.8)	−2.6%
Tobacco	Smoking	12.6 (8.8, 17.3)	24.7 (17.7, 33.2)	3.3%	0.3 (0.2, 0.4)	0.2 (0.2, 0.3)	−0.8%
	Secondhand smoke	59.7 (24.6, 92.5)	117.1 (46.1, 188)	3.3%	1.5 (0.6, 2.3)	1.2 (0.5, 1.9)	−0.7%
Dietary factors	Diet low in fruits	30.3 (18.2, 43.7)	33.4 (12.9, 60.4)	0.4%	0.8 (0.4, 1.1)	0.3 (0.1, 0.6)	−1.9%
	Diet low in fiber	12.4 (4.8, 20.7)	15.5 (4.7, 28.6)	0.9%	0.3 (0.1, 0.5)	0.2 (0, 0.3)	−1.7%
	Diet low in whole grains	18.2 (4.8, 28.9)	41.8 (12.6, 66.9)	4.5%	0.5 (0.1, 0.7)	0.4 (0.1, 0.7)	−0.3%
	Diet low in nuts and seeds	9.3 (1.2, 19.8)	16.5 (1.9, 38.6)	2.7%	0.2 (0, 0.5)	0.2 (0, 0.4)	−1.0%
	Diet high in red meat	22.7 (8.8, 35.9)	80.7 (47.7, 117)	8.8%	0.6 (0.2, 0.9)	0.8 (0.5, 1.1)	1.5%
	Diet high in processed meat	6.3 (3.3, 8.7)	26.5 (12.6, 37.3)	11.1%	0.2 (0.1, 0.2)	0.3 (0.1, 0.4)	2.3%
	Diet high in sugar-sweetened beverages	13 (9.4, 17.3)	21.8 (12.7, 30.5)	2.3%	0.3 (0.2, 0.4)	0.2 (0.1, 0.3)	−1.1%
**Males**							
Lifestyle	High BMI	40.3 (10, 88.4)	228.3 (105, 383.5)	16.1%	1 (0.2, 2.3)	2.5 (1.1, 4.2)	5.0%
	Low physical activity	14 (6, 26.1)	48 (19.8, 92.3)	8.4%	0.6 (0.3, 1.1)	0.7 (0.3, 1.4)	0.8%
Environmental pollutants	Ambient particulate matter pollution	23.1 (10, 39.9)	162.4 (110.1, 220.3)	20.8%	0.7 (0.3, 1.2)	2.1 (1.4, 2.8)	6.4%
	Household air pollution from solid fuels	43 (25.9, 64.1)	32.8 (14.9, 61.2)	−0.8%	1.3 (0.8, 1.9)	0.4 (0.2, 0.7)	−2.4%
Tobacco	Smoking	70.2 (56.4, 85.9)	193.7 (147.2, 244.9)	6.1%	1.9 (1.6, 2.3)	2.2 (1.7, 2.8)	0.6%
	Secondhand smoke	21.3 (7.8, 35.2)	63.4 (24.4, 103.2)	6.8%	0.7 (0.3, 1.1)	0.8 (0.3, 1.3)	0.7%
Dietary factors	Diet low in fruits	21.2 (12.9, 30.9)	35.2 (13.1, 63)	2.3%	0.6 (0.4, 0.9)	0.5 (0.2, 0.8)	−1.0%
	Diet low in fiber	8.2 (3.2, 13.7)	13.3 (4.1, 24.4)	2.1%	0.3 (0.1, 0.4)	0.2 (0.1, 0.3)	−1.1%
	Diet low in whole grains	13.3 (4.2, 21)	42 (12.7, 67.5)	7.5%	0.4 (0.1, 0.6)	0.5 (0.2, 0.8)	1.0%
	Diet low in nuts and seeds	6.5 (0.7, 14.3)	15.4 (1.8, 35.3)	4.8%	0.2 (0, 0.4)	0.2 (0, 0.4)	−0.1%
	Diet high in red meat	16.5 (7.4, 25.6)	80.3 (48, 116.3)	13.3%	0.5 (0.2, 0.7)	1 (0.6, 1.4)	3.4%
	Diet high in processed meat	4 (2.2, 5.5)	22.8 (10, 32.1)	15.9%	0.1 (0.1, 0.2)	0.3 (0.1, 0.4)	4.1%
	Diet high in sugar-sweetened beverages	10.4 (7.9, 13.2)	23.6 (15.4, 32.2)	4.4%	0.3 (0.2, 0.4)	0.3 (0.2, 0.4)	−0.1%
**GBD 2019 risk factors in China**		**All-ages DALYs number (×104, 95% CI)**			**Age-standardized DALYs rate (/100,000, 95% CI)**		
		**1990**	**2019**	**Annual growth%**	**1990**	**2019**	**Annual growth%**
**Females**							
Lifestyle	High BMI	41.7 (12.1, 84.4)	177.1 (92.9, 281.8)	11.2%	88.8 (25.7, 178.5)	167.7 (86.9, 0)	3.1%
	Low physical activity	11.9 (5.3, 21.9)	27.1 (12.2, 50.2)	4.4%	28.7 (13, 51.8)	26.5 (12.1, 0)	−0.3%
Environmental pollutants	Ambient particulate matter pollution	13.2 (5.7, 24.5)	82.7 (54.1, 114.6)	18.1%	29.6 (12.9, 54.8)	79.1 (51.8, 0)	5.8%
	Household air pollution from solid fuels	37.5 (23.2, 57.2)	23.9 (11.8, 41.6)	−1.3%	83.7 (51.8, 127.2)	22.8 (11.3, 0)	−2.5%
Tobacco	Smoking	5.9 (4, 8.1)	12.4 (8.9, 16.7)	3.9%	13.4 (9.3, 18.5)	11.7 (8.4, 0)	−0.4%
	Secondhand smoke	33.9 (13.3, 53.1)	68.7 (25.4, 110.1)	3.5%	74.8 (29.6, 116.8)	65.6 (24.3, 0)	−0.4%
Dietary factors	Diet low in fruits	16.5 (9.9, 24.3)	17.8 (6.8, 32.7)	0.3%	36.8 (22, 54.1)	17.3 (6.8, 0)	−1.8%
	Diet low in fiber	7 (2.9, 11.8)	9 (2.9, 16.7)	1.0%	15.6 (6.4, 26.1)	8.7 (2.8, 0)	−1.5%
	Diet low in whole grains	9.7 (2.7, 15.9)	22.3 (6.4, 37)	4.5%	21.8 (6.1, 35.6)	21.4 (6.1, 0)	−0.1%
	Diet low in nuts and seeds	5 (0.6, 10.7)	8.5 (1, 19.1)	2.4%	11.1 (1.4, 24)	8.1 (0.9, 0)	−0.9%
	Diet high in red meat	12.8 (5.4, 20.2)	47.5 (29.4, 69)	9.3%	28.2 (11.8, 44.6)	45.2 (28, 0)	2.1%
	Diet high in processed meat	3.5 (1.7, 5)	16.7 (8.1, 23.9)	12.8%	7.8 (3.8, 11)	15.9 (7.8, 0)	3.6%
	Diet high in sugar-sweetened beverages	6.7 (4.7, 9.2)	11.7 (6.5, 16.8)	2.5%	15.2 (10.7, 20.5)	11.2 (6.3, 0)	−0.9%
**Males**					0 (0, 0)	0 (0, 0)	0.0%
Lifestyle	High BMI	35.5 (8.8, 77)	196.7 (100.1, 310.9)	15.7%	71.5 (17.6, 156.5)	195.1 (99.3, 0)	6.0%
	Low physical activity	6.2 (2.4, 11.8)	18.1 (7, 36.8)	6.7%	18.8 (7.8, 34.4)	21 (8.4, 0)	0.4%
Environmental pollutants	Ambient particulate matter pollution	15.9 (6.8, 28.1)	98.9 (65.4, 135.1)	18.0%	36.1 (15.5, 63.8)	101.6 (67.8, 0)	6.3%
	Household air pollution from solid fuels	30.3 (17.3, 46.2)	19.5 (8.7, 35.7)	−1.2%	68.3 (39.5, 104.5)	19.8 (8.8, 0)	−2.4%
Tobacco	Smoking	51.2 (39.4, 66.6)	129.6 (94.7, 165.5)	5.3%	111.4 (86.7, 144.2)	127.8 (93.9, 0)	0.5%
	Secondhand smoke	14 (4.9, 23.2)	36.2 (13.6, 60.3)	5.5%	32.5 (11.5, 53.6)	37.2 (14, 0)	0.5%
Dietary factors	Diet low in fruits	15.4 (9.1, 23.3)	22 (8.6, 40.4)	1.5%	34.4 (20.4, 51.4)	23 (9.4, 0)	−1.1%
	Diet low in fiber	6.3 (2.5, 10.3)	9.3 (3, 16.7)	1.6%	13.9 (5.5, 23.1)	9.6 (3.2, 0)	−1.1%
	Diet low in whole grains	9.3 (2.7, 15.4)	25.7 (7.6, 41.5)	6.1%	21 (6.2, 34.5)	26.3 (7.7, 0)	0.9%
	Diet low in nuts and seeds	4.5 (0.5, 9.9)	9 (1, 20.3)	3.5%	10.2 (1.2, 22.3)	9.4 (1.1, 0)	−0.3%
	Diet high in red meat	12.2 (5.6, 19.5)	54.2 (33.6, 76.6)	11.8%	26.8 (12.3, 42.2)	54.6 (33.6, 0)	3.6%
	Diet high in processed meat	3 (1.5, 4.3)	16.3 (7.5, 23.8)	15.3%	6.6 (3.4, 9.4)	16.4 (7.5, 0)	5.1%
	Diet high in sugar-sweetened beverages	7 (5.1, 9.4)	14.2 (8.7, 20)	3.5%	16.1 (11.9, 21.2)	14.8 (9.1, 0)	−0.3%

### The SEV of T2DM attributable risks in China

Estimated by SEV, the leading five risk factors were a diet low in whole grain (83.11, 95% UI: 77.65–94.94), a diet low in red meat (70.70, 95% UI: 61.87–78.16), secondhand smoke (49.33, 95% UI: 47.23–51.32), diet low in fruits (45.26, 95% UI: 34.85–57.18), and diet low in nuts and seeds (44.38, 95% UI: 18.62–66.42). HBMI, APMP, and a diet high in processed meat were the leading three risks with the greatest change in these 30 years. Although HBMI has been a key attributable risk factor, the SEV has been only 14.08 (95% UI: 9.79–19.46) in males and 12.20 (95% UI: 8.03–17.77) in females. Considering SEV, household air pollution from solid fuels has achieved the sharpest downward shifts in ranking overall. Estimated by SEV attributable for tobacco in 2019, smoking has been 27.42 (95% UI: 24.66–30.37) in males, nearly 16 times that of females (1.76, 95% UI: 1.49–2.05). Whereas secondhand smoke in females was 63.39 (95% UI: 60.83–65.83), nearly doubled in males (35.71, 95% UI: 32.57–38.51). A lower degree of SEV and a higher level of attributable T2DM-related burden could be found in HBMI, APMP, and smoking, meaning a critical role of them in the development and progression of T2DM (Supplementary Figure S1).

### The relationship between the disease burden of T2DM and SDI in China

**Figure 4:**
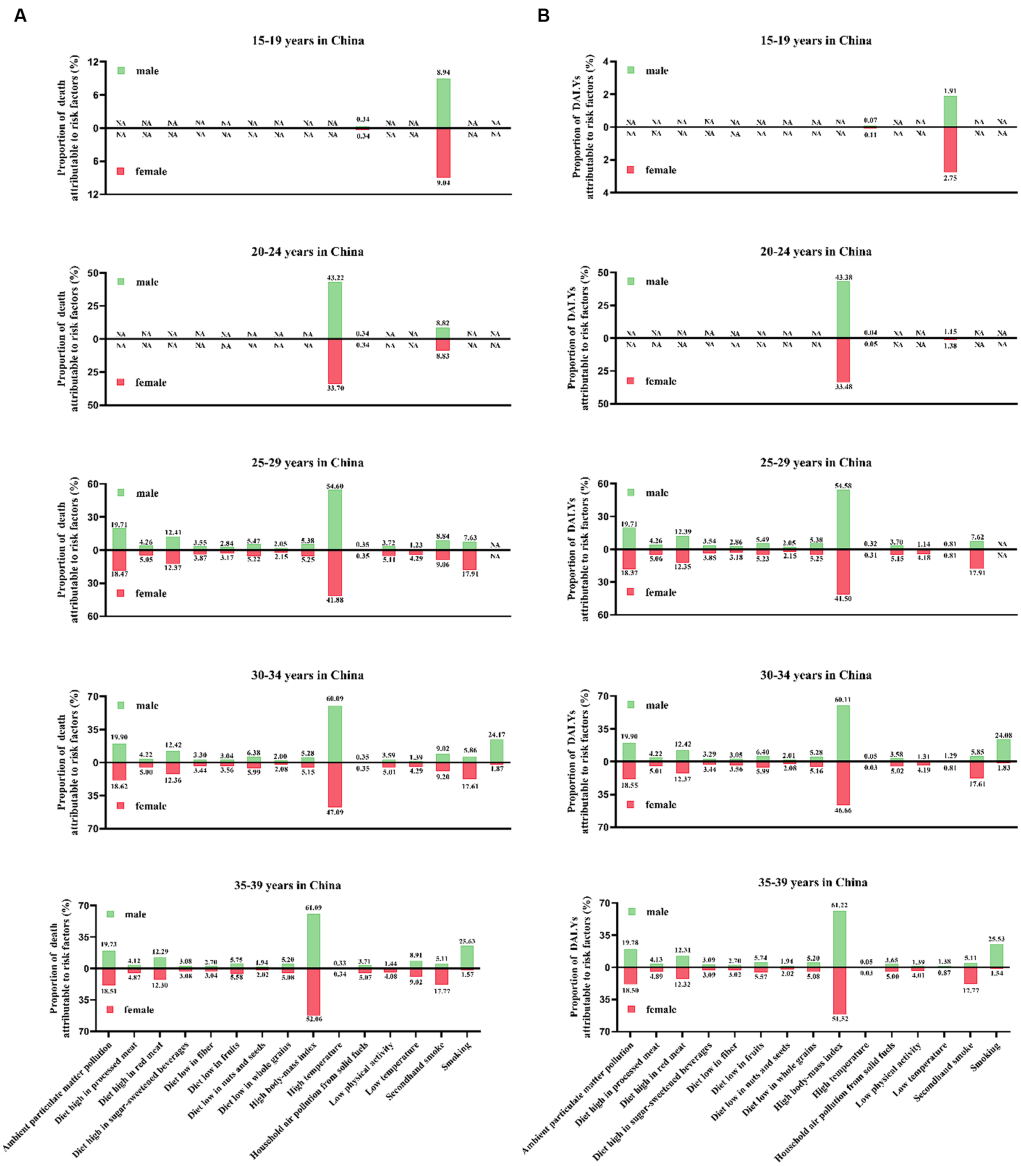
The proportion of the T2DM-related burden attributable to 15 risk factors in 2019 by different age groups. **(A)** Deaths and **(B)** DALYs.

**Figure 5:**
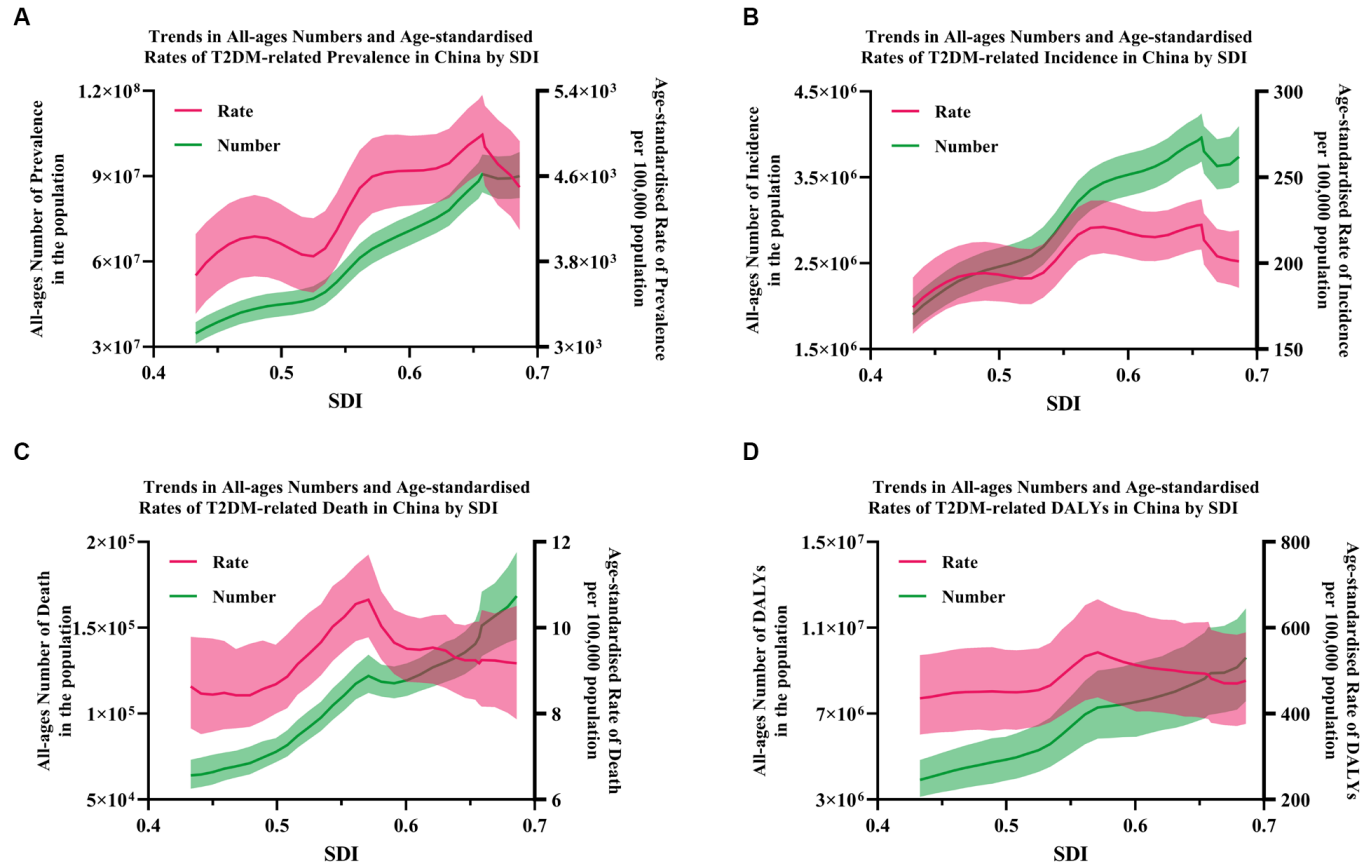
The correlation between the burden of T2DM and SDI in China. **(A)** Prevalence; **(B)** incidence; and **(C)** death; **(D)** DALYs. Shading indicates the 95% UI.

HBMI has been the key attributable risk factor for all different developmental stages in China. However, the contributions of different risks varied across SDI. By SDI, we found the highest age-standardized incidence and DALYs rate in stages with low–middle-SDI stages, and in low-SDI stages, we had the lowest age-standardized incidence rate but the highest age-standardized mortality rate. In China, an inverse U-shaped curve could be found in the association between age-standardized incidence, mortality, and DALYs rate and SDI, meaning in low–middle and middle-SDI countries owe a higher age-standardized incidence and DALYs rate, and higher age-standardized mortality rate in low-SDI countries. Moreover, the SDI level was positively associated with faster increases in the age-standardized incidence and DALYs rate of T2DM but with faster reductions in the age-standardized mortality rate. We found that when SDI was associated with mortality and DALYs rate, an inverse U-shaped curve was observed between T2DM-related disease burden and income tier of China. To be specific, the low and lower-middle-income development stages were mostly environment-related, while the upper-middle-income and high-income development stages were mostly lifestyle-related (Figures 4, 5; Supplementary Figures S3–S5).

## Discussion

**Figure 6:**
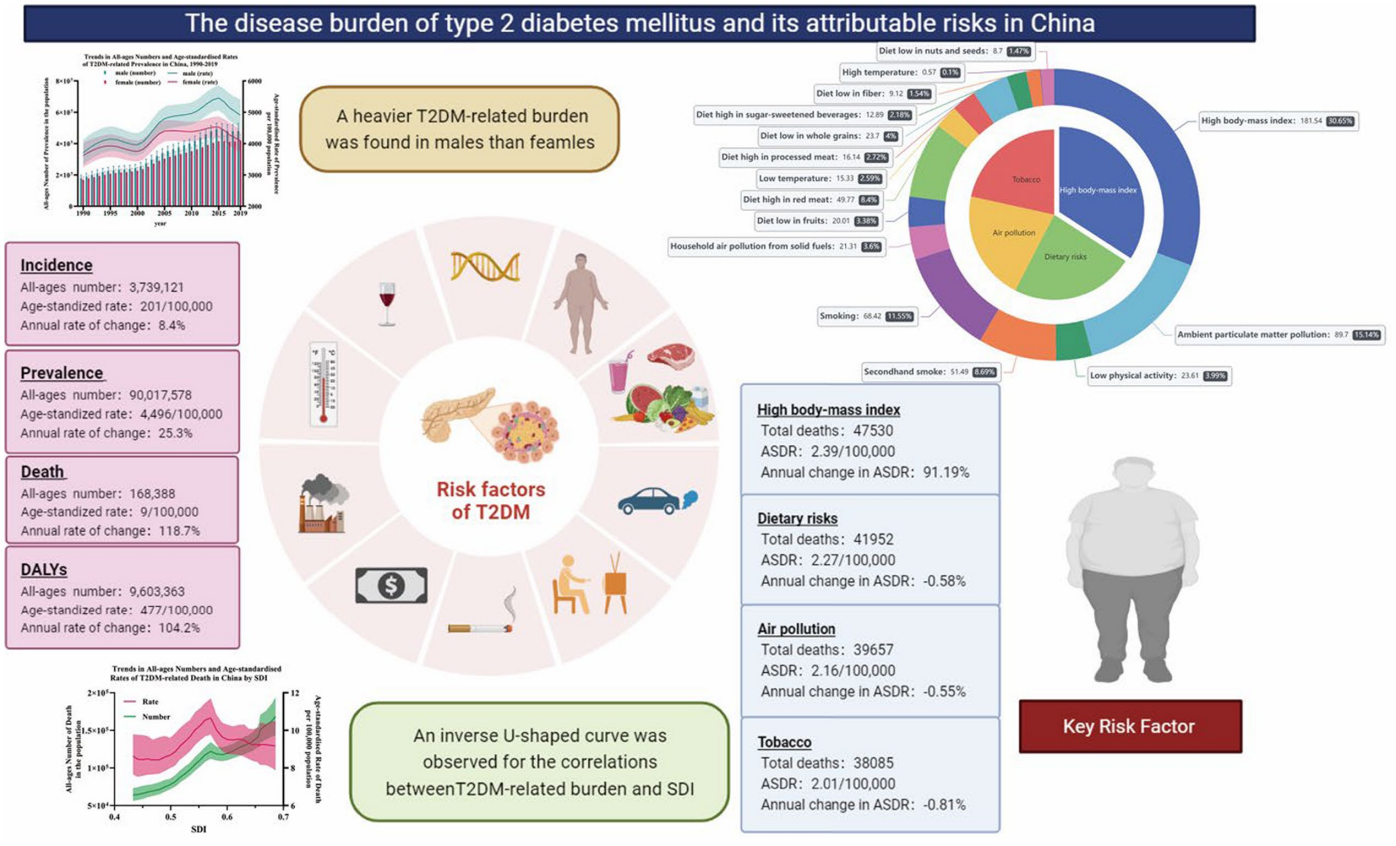
The main findings of the study.

Our study provides insights into the trends of the disease burden of T2DM and its attributable risk factors in China from 1990 to 2019. A relatively detailed descriptive analysis has been performed using the four epidemiological indicators (prevalence, incidence, mortality, and DALYs). A second analysis was applied to gender disparities, different age distributions and inconsistent socioeconomic levels. Generally, the number of deaths and DALY attributable to T2DM nearly tripled from their 1990 levels to 168.4 thousand and 9.6 million in 2019. Age-standardized death rate and age-standardized DALY rate also increased by 7.0 and 9.3% to 9.2 and 476.5 per 100,000 population during the same period. A single-peak distribution has been found in the trends of the number of T2DM-related burdens by age. However, the curve of mortality and DALYs rates rose significantly with age, implying that population aging may be the critical factor for the phenomenon. Considering the annual growth rate of T2DM-related burden, significant increases in all-ages numbers but only slight rises in age-standardized rates have been observed, which may partly be due to the population expansion. Totally, a heavier disease burden could be found in males. Benefiting from the protective effects of estrogen prior to menopause, males showed a higher number of deaths and DALYs than females until nearly age 62. Partly due to the higher life expectancy of females, higher numbers but lower rates of deaths and DALYs could be found in postmenopausal females. Among these risk factors, the top five were HBMI, APMP, smoking, low physical activity, and secondhand smoke. The key risk factors in China were HBMI and APMP, which accounted for 47.11% of deaths and 57.8% of DALYs (Figure 6).

Our research indicated that the steep rise of T2DM has gone along with gender differences. The difference in gender distribution of the disease burden of T2DM is closely related to the diversities in physiological and metabolic factors, lifestyle, education levels, socioeconomic and cultural factors, genetic effects, epigenetic mechanisms, etc. In terms of physiological metabolism, relevant studies have shown that under the same BMI, men have significantly higher visceral fat than women, which is significantly related to metabolic syndrome and cardiovascular disease (24). Moreover, the quality and activity of brown adipose tissue are also higher in women, which is closely related to insulin resistance, energy metabolism and obesity (25). Thus, males are usually diagnosed with diabetes at younger ages and have lower BMI than women (26). Recent meta-analyses have shown that males have lower leptin and adiponectin levels than females of comparable age and BMI, which are important in the regulation of food intake, satiety, and energy expenditure (27–29). In addition, males are more inclined to misperceive their weight control and healthy nutrition, resulting in low physical activities, consuming more red meat, processed meat, or sugar-sweetened beverages and less fruits, whole grains, or vegetables (30, 31). In addition, with greater social pressure and financial burden in males, a higher prevalence of smoking and overdrinking was observed in them (13). A 13-year follow-up study found that affected by social attributes and family division of labor, women pay more attention to the disease, resulting in higher treatment compliance and better blood sugar self-management (32).

We deeply explored the association between socioeconomic development and the disease burden by analyzing epidemiological data, including incidence, prevalence, mortality, and DALYs of T2DM in different income levels and different SDI stages in China. After standardization, the disease burden of T2DM and SDI showed an inverted U-shaped relationship. The disease burden is heaviest in low- and middle-income stages in China, with rapid growth and huge numbers, nearly tripling compared to 1990, indicating that the situation of T2DM in economically underdeveloped developing countries is still serious and cannot be ignored. Although the incidence and prevalence of T2DM are still at a high level in China with higher income levels, the mortality and DALYs rates are significantly lower than those at other income levels, indicating that in more economically developed countries, patients with T2DM with high levels of screening, diagnosis and timely treatment, the prognosis is better, and the actual disease burden is lighter. The results of a large-scale epidemiological survey by the American Diabetes Association (ADA) in 2017 showed that the awareness rate, treatment rate, and control rate of the disease among patients with diabetes are still very low (36.5, 32.2, and 49.2%, respectively). The proportion of undiagnosed diabetes is still high (28%), especially in economically underdeveloped areas or rural areas (33, 34). A recent study in India showed that more than 50% of diabetic patients among people aged 15–50 have not been diagnosed, and about 42% of patients have no awareness of diabetes (35). Recent meta-analyses have shown that educated patients have stronger self-management abilities; usually indicators such as blood sugar, blood lipids, and blood pressure are better controlled, the incidence of complications is lower, and treatment costs are less (36, 37). The IDF 2021 report points out that 75% of basal insulin is provided by the government to children in high-income countries, but 50% in middle-income countries, and none in low-income countries. Basic treatment drugs, such as metformin, are readily available (88%) in high-income countries but 64% in middle-income countries and 20% in low-income countries (38, 39). The same situations could be found in a limited supply of advanced equipment such as continuous blood glucose monitoring equipment and insulin pump (39). SDI is a comprehensive measure of socioeconomic development based on three aspects: the average national education level of the population over 15 years old, the total fertility rate of the population under 25 years old, and *per capita* income. The higher the SDI level, correspondingly, the medical and health resources are more sufficient, the transportation is more convenient, the residents are often more educated, the T2DM screening system is more complete and more easily accepted by the people, early diagnosis, and the rate of diagnosis is higher. Considering the development of China, timely and standardized intervention and treatment can significantly improve the prognosis of patients with T2DM. Increasing antidiabetic drug reserves and introducing advanced monitoring equipment and technology are particularly important in these countries.

Over the past 28 years, rapid development, population aging and lifestyle transitions have had a huge impact on public health. As indicated in our study, HBMI was the key risk factor for T2DM-related deaths and DALYs in China and across all age groups. Obesity is a well-documented important public health issue and a key contributor to numerous chronic diseases, including cardiovascular diseases (ischemic heart disease, stroke, and hypertensive heart disease), diabetes mellitus, chronic kidney diseases, neoplasms, neurological disorders, and chronic respiratory diseases. One GBD study showed that 16.7% of global cardiovascular disease cases, 28.6% of global diabetes and kidney disease cases, and 4.8% of global cancer cases in 2017 were attributable to HBMI (40). Taking a rapidly developing country like India as an example, the overweight rate in India increased from 9.0% in 1990 to 20.4% in 2016, and 36.0% of T2DM DALYs could be attributed to HBMI (41). An upward trend could also be found in China; the overweight rate and obesity rate among adults aged 18 and over nationwide were 30.1 and 11.9% in 2015, respectively, showing an increase of 7.3 and 4.8% from 2002 (42). The increasingly heavier burden of obesity prevalence was undoubtedly associated with economic development and lifestyles’ change. Lifestyle transitions, including increased high-calorie foods and sweetened-beverage consumption, changes in the food environment and industry, agricultural practices, and the implementation of various food and drink restrictions and taxation, all resulted in considerable shifts in dietary patterns (43, 44). Thanks to the boom of transportation, ultra-processed, highly energy-dense, and rich in fat, salt, and glycemic load, foods have become more and more available and affordable. In addition, urbanization, together with low physical activity and a sedentary lifestyle, increased time spent watching television or playing video games, to a large extent have become another driver of overweight or obesity (45). As demonstrated by the US Diabetes Prevention Program (46), the Dutch Diabetes Prevention Study (47), and the American Cancer Society’s Cancer Prevention Study (48), all reported that intentional weight loss was associated with the reduction in T2DM-related mortality and its complications. Participants with >5% weight loss had a 64% lower risk of developing diabetes during the first 6 years than the follow-up, whereas those with 3–5% weight loss also had a 40% lower risk (46, 47). Therefore, the government should pay attention to the health benefits brought by weight management, encourage relevant media to publicize correct weight management models and metabolic goals, and display successful cases on major APP platforms. Relevant studies have shown that dietary patterns such as the Mediterranean diet, vegetarian diet, low-carbohydrate diet, and low-fat and low-energy diet are helpful for weight control. However, in practice, it should still be completed under the guidance of professionals, considering the patient’s personal situation, such as metabolic goals, economic status, social factors, etc., and developing individualized dietary patterns and testing follow-up plans.

As reported by population-based studies, statistically significant increased mortality risks could be found in the relationships between exposure to air pollution and diabetes [e.g., a 10-μg/m3 increment in PM2.5 was associated with a 6–49% increase in diabetes-related mortality]. The higher levels of PM2.5 and nitrogen dioxide, the heavier T2DM mortality (49). According to a meta-analysis, a 25–40% decrease in the risk of T2DM could be found in most activities, including leisure-time activity (20%), resistance exercise (30%), occupational activity (15%), cardiorespiratory fitness (55%), and walking (15%) (35). Studies have shown that men who smoke more than two packs of cigarettes per day have a 45% higher risk of developing diabetes than non-smoking men, while in women, it was even 75% (50). Nurses’ Health Study in the United States showed that after adjustment for other risk factors, the risk for diabetes in smokers was 1.42 (51). The most recent research indicates that with each 250-ml/d increase in sugar-sweetened beverages and artificially sweetened beverages intake, the risk increased by 12 and 21% for obesity, 19 and 15% for T2DM, 10 and 8% for hypertension, and 4 and 6% for all-cause mortality (52). Several mechanisms such as elevated oxidative stress and systemic inflammation, impaired endothelial function, endoplasmic reticulum stress, as well as cardiac autonomic nervous system and mitochondrial dysfunction, have been proposed to explicate the phenomenon. Therefore, the government should provide subsidies and incentives for the consumption of healthy foods such as whole grains, nuts, fiber, fruits, and vegetables (53). Higher taxes should be used in foods with high sugar and salt content, such as sauces and pickled products (54). Health programs and related media should emphasize the benefits of healthy dietary patterns and stress the importance of a reasonable nutrient composition ratio. At the same time, more attention should be paid to the training of nutritional therapists. Efforts should be made to equip the outpatient and inpatient departments of the medical system with at least one nutritional therapist to provide nutrition education and management to patients and their families. The popularization of new energy and natural gas is particularly important for air pollution. Purchasing new energy vehicles should be supported by policies and given certain discounts to buyers. In addition, strengthening air pollution control is also very important in countries in industrialization transition and countries with heavy manufacturing industry transfers. These countries or areas should pay attention to environmental management while developing. Following the World Health Organization’s Framework Convention on Tobacco Control, managers should formulate a comprehensive smoke-free policy, use health warning labels and anti-tobacco mass media campaigns, ban tobacco advertising, promotion and sponsorship, increase taxes on tobacco products and raise tobacco prices to reduce the consumption of Tobacco. The medical system should add smoking cessation clinics to provide regular education to smokers, inform smokers of the dangers of smoking and secondhand smoke, and the benefits of quitting smoking, open a smoking cessation hotline, and provide free individualized plans and follow-up plans for those who intend to quit smoking, and provide free psychological counseling during the smoking cessation process.

A strength of our study was that we had estimated the disease burden of T2DM and its attributable risk factors in China, measured with comprehensive indicators (DALYs, SEV, and SDI), meanwhile linked with gender difference, different age groups, and inconsistent socioeconomic development. These findings provide insight into how weight control could reduce the risk of T2DM to a great extent. Just like ‘Healthy China 2030’, declared in 2016, aims to facilitate appropriate diet and adequate physical activity to reduce obesity and lower the incidence of T2DM. As the largest developing country, China has been undergoing social transformation, rapid development, population aging, and lifestyle transitions. Other countries could find their own shadow in China. Therefore, neighboring countries, especially middle and low-middle-income developing countries, could use it for reference to enable policymakers to tailor their policies to targeted control and adjust preventative strategies, considering their levels of economic development and their leading adjustable risks.

This study also has some limitations. First, the GBD 2019 study provided estimates using hierarchical models in representative population-based studies, whose original data sources and statistical processing may introduce bias. Second, many risk factors are co-influencers, so it is difficult to distinguish the independent effects of single risk exposure. Third, different health information systems in different countries and regions also contain information gaps. For example, Asians generally had a higher percentage of body fat than white people of the same age, gender, and BMI, so using the same cut-off point might underestimate the related risks in the Asian population.

In conclusion, the total disease burden of T2DM in China has increased significantly with time. Gender disparities and different age distributions could be found in it. The most growing burden of T2DM could be attributed to modifiable risk factors, especially in HBMI, APMP and tobacco. Therefore, policymakers should focus on weight loss and environmental protection. Considering the T2DM disease burden trends in China, neighboring countries could learn from them to suit targeted control and preventative strategies with their local conditions (economy levels and leading adjustable risks) to soar the burden.

## Data availability statement

The original contributions presented in the study are included in the article/Supplementary material, further inquiries can be directed to the corresponding author.

## Ethics statement

The studies involving humans were approved by the GBD 2019 https://vizhub.healthdata.org/gbd-results/. The studies were conducted in accordance with the local legislation and institutional requirements. Written informed consent for participation was not required from the participants or the participants’ legal guardians/next of kin in accordance with the national legislation and institutional requirements.

## Author contributions

XH: Conceptualization, Data curation, Formal analysis, Investigation, Software, Visualization, Writing – original draft, Writing – review & editing. YH: Data curation, Investigation, Funding acquisition, Writing – review & editing. HX: Data curation, Funding acquisition, Writing – review & editing. YS: Methodology, Resources, Writing – review & editing. XP: Data curation, Supervision, Writing – review & editing. JW: Investigation, Validation, Visualization, Writing – review & editing. KC: Data curation, Writing – review & editing.

## Footnotes

^1^ https://vizhub.healthdata.org/gbd-results/
