# Supplementary material for: Association between sociodemographic status and the T2DM-related risks in China: implication for reducing T2DM disease burden
Source: Front Public Health. 2024 Jan 8;11:1297203. doi: 10.3389/fpubh.2023.1297203 (PMC10801005; doi:10.3389/fpubh.2023.1297203)
Supplement: Supplementary file 1 [file Presentation_1.pdf]

The supplementary captions with supplementary images

A

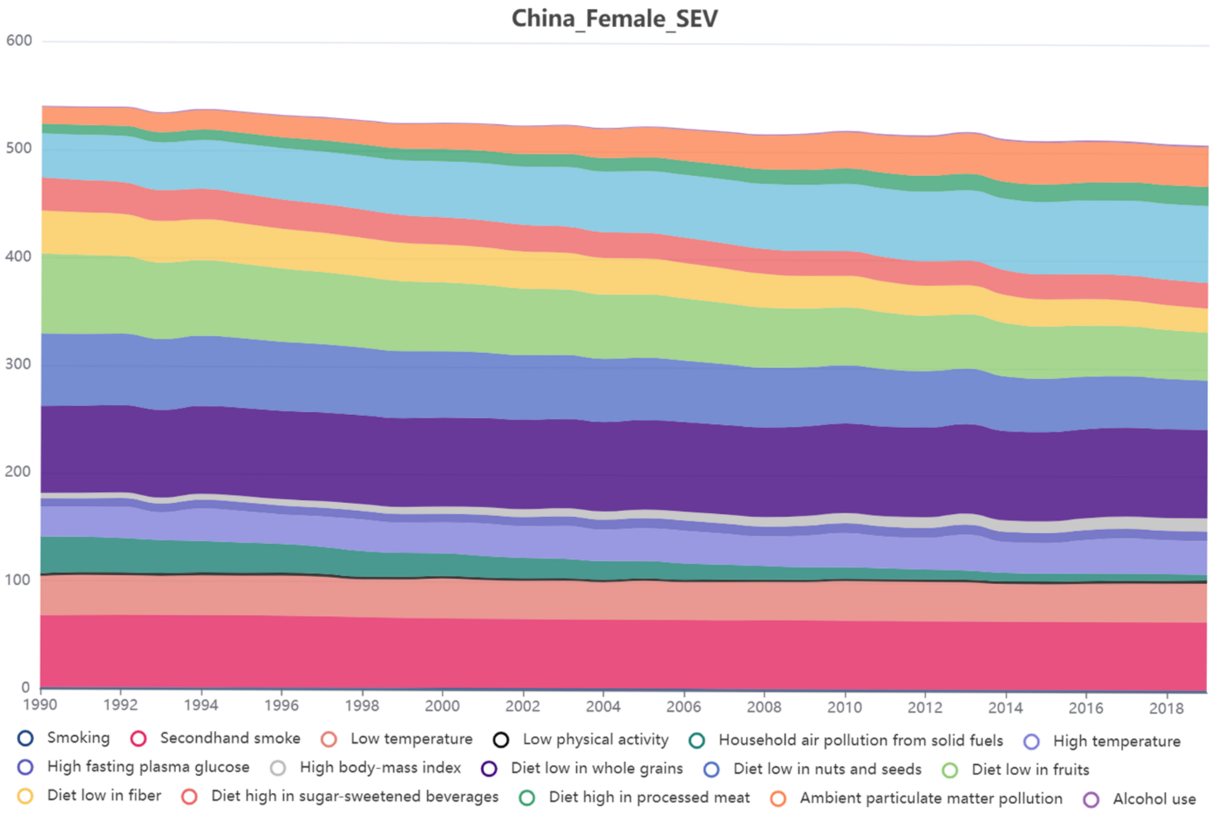
China_Female_SEV
600
500
400
300
200
100
0
1990 1992 1994 1996 1998 2000 2002 2004 2006 2008 2010 2012 2014 2016 2018
Smoking
Secondhand smoke
Low temperature
Low physical activity
Household air pollution from solid fuels
High temperature
High fasting plasma glucose
High body-mass index
Diet low in whole grains
Diet low in nuts and seeds
Diet low in fruits
Diet low in fiber
Diet high in sugar-sweetened beverages
Diet high in processed meat
Ambient particulate matter pollution
Alcohol use

B

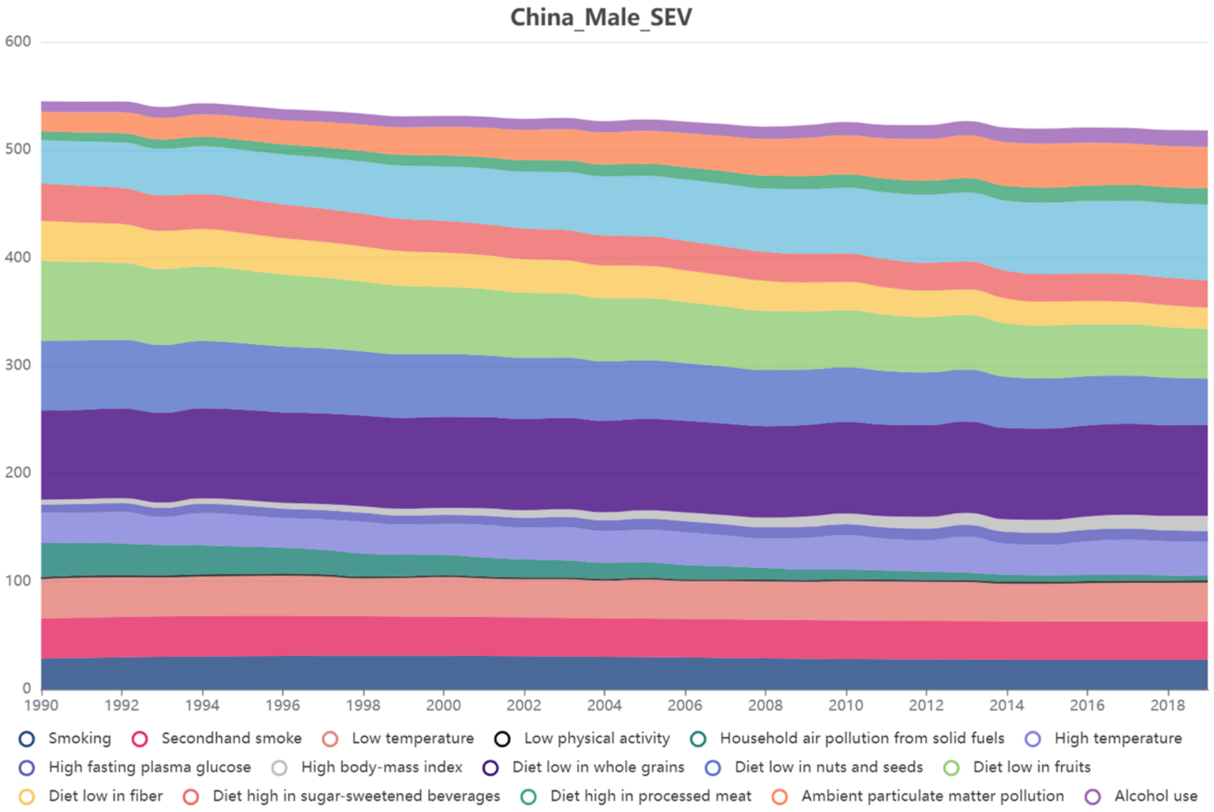
China_Male_SEV
600
500
400
300
200
100
0
1990 1992 1994 1996 1998 2000 2002 2004 2006 2008 2010 2012 2014 2016 2018
Smoking
Secondhand smoke
Low temperature
Low physical activity
Household air pollution from solid fuels
High temperature
High fasting plasma glucose
High body-mass index
Diet low in whole grains
Diet low in nuts and seeds
Diet low in fruits
Diet low in fiber
Diet high in sugar-sweetened beverages
Diet high in processed meat
Ambient particulate matter pollution
Alcohol use

Supplementary Figure 1. The SEV of the most detailed risk factors attributable to T2DM-related burden from 1990 to 2019 in China in different gender. **(A)** Female; **(B)** Male.

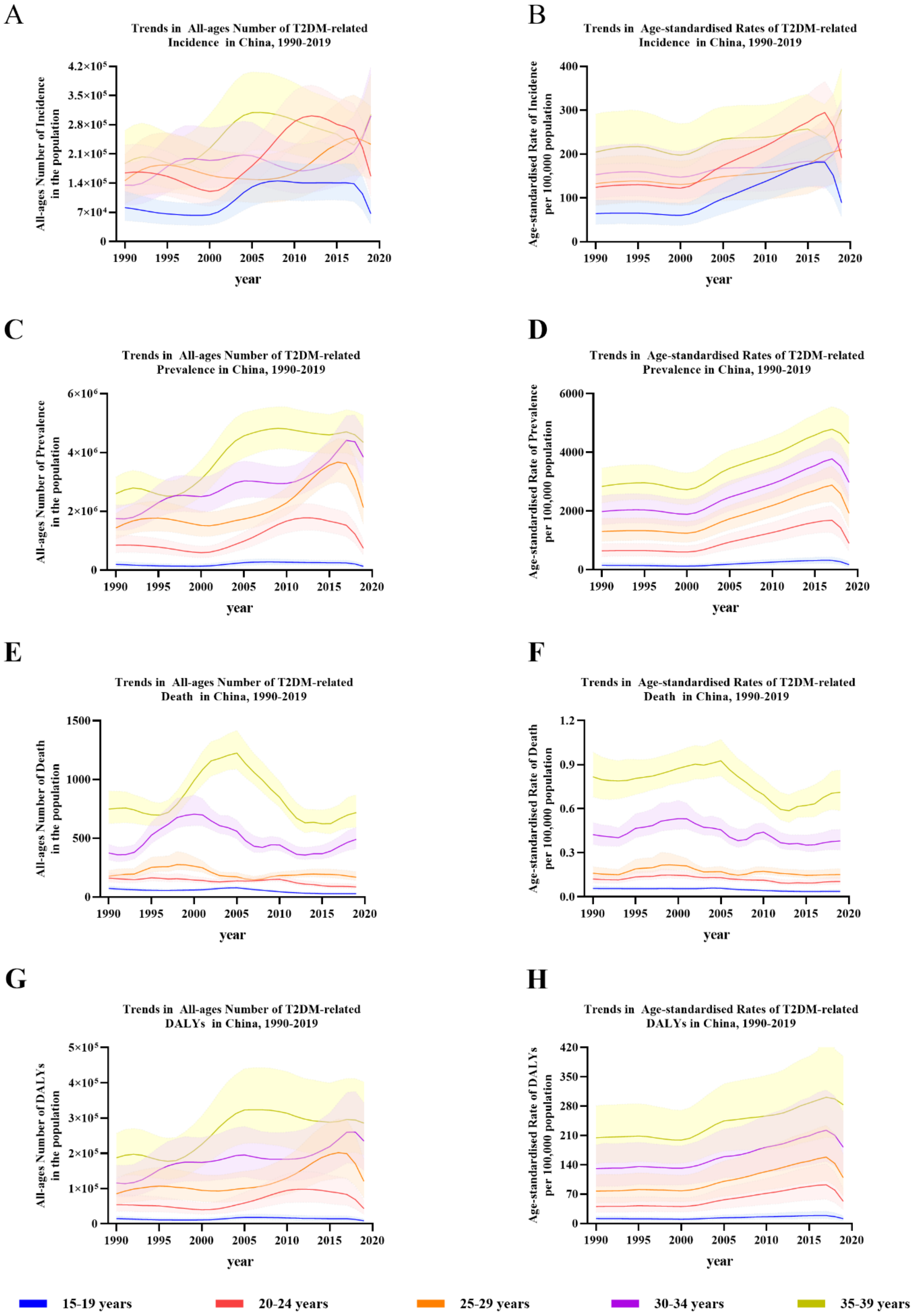

Shading indicates the 95% uncertainty interval (UI) for all-ages numbet and age-standardised rate

Supplementary Figure 2. The burden of T2DM from 1990 to 2019 in China with different young age groups. **(A)** Trends in All-ages Numbers of T2DM-related Incidence in China, 1990-2019; **(B)** Trends in Age-standardized Rates of T2DM-related Incidence in China, 1990-2019; **(C)** Trends in All-ages Numbers of T2DM-related Prevalence in China, 1990-2019; **(D)** Trends in Age-standardized Rates of T2DM-related Prevalence in China, 1990-2019; **(E)** Trends in All-ages Numbers of T2DM-related Death in China, 1990-2019; **(F)** Trends in Age-standardized Rates of T2DM-related Deaths in China, 1990-2019; **(G)** Trends in All-ages Numbers of T2DM-related DALYs in China, 1990-2019; **(H)** Trends in Age-standardized Rates of T2DM-related DALYs in China, 1990-2019. Shading indicates the 95% uncertainty interval (UI) for rates.

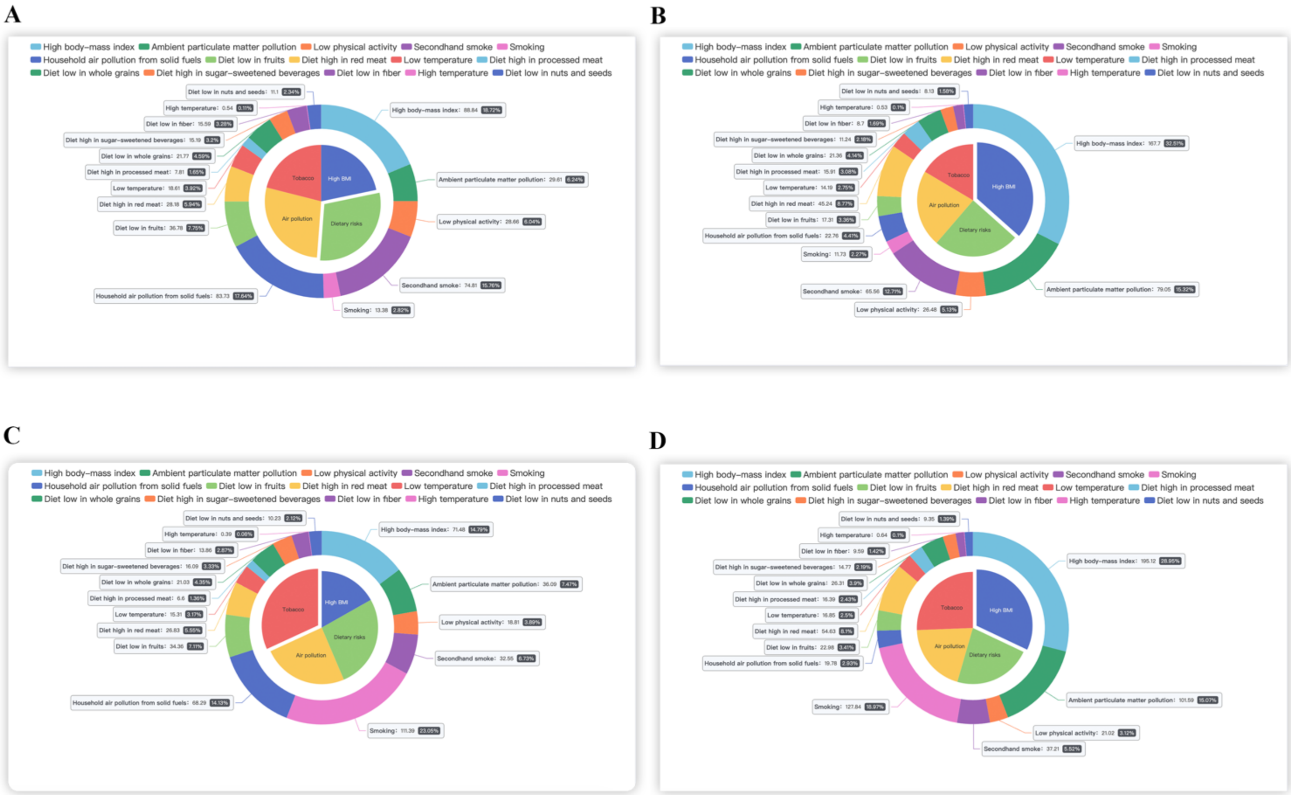

Supplementary Figure 3. Proportion of the T2DM-related DALYs attributable to 15 risk factors in 1990 and 2019 by different gender. **(A)** females in 1990; **(B)** females in 2019; **(C)** males in 1990; **(D)** males in 2019.

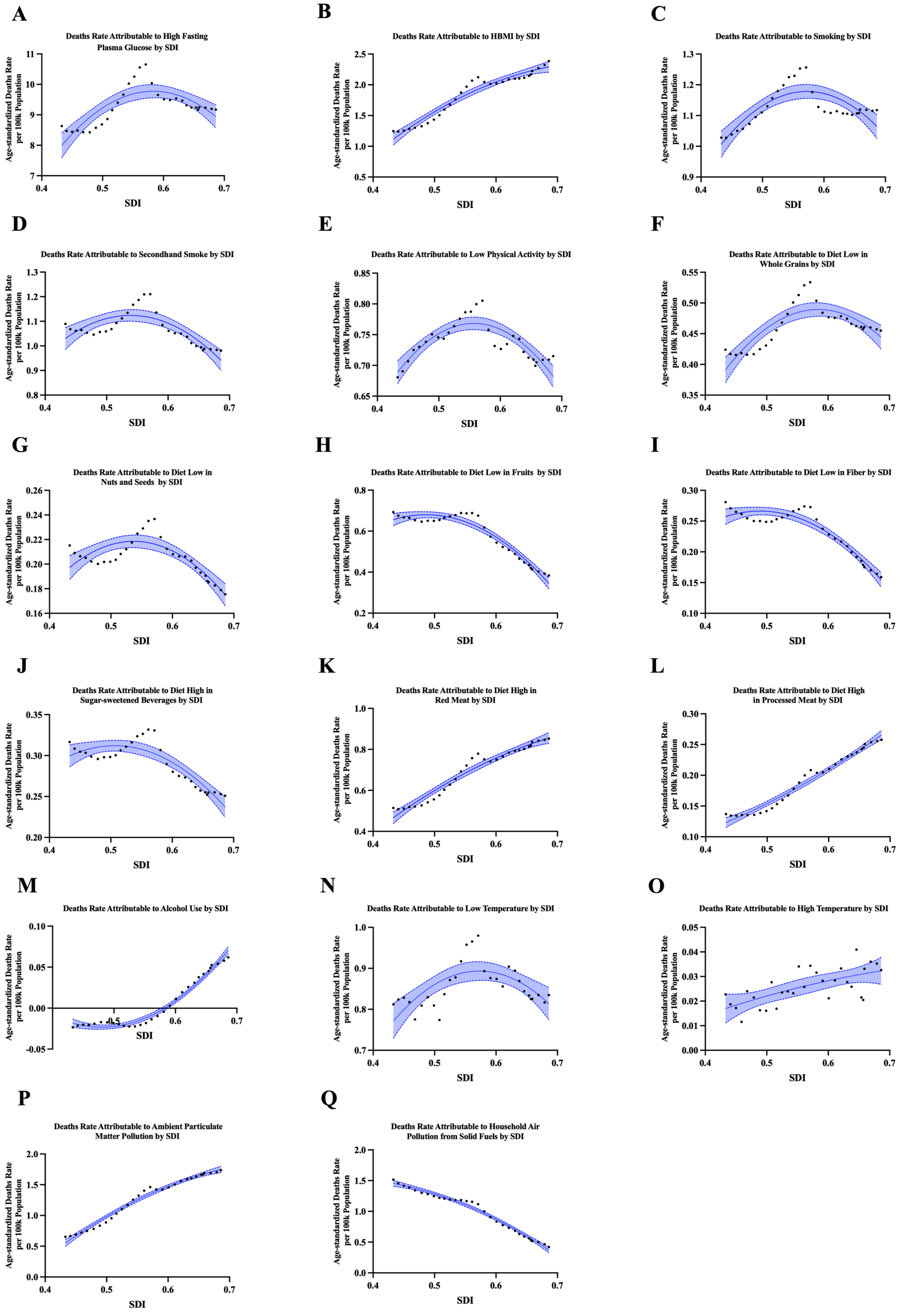
A
Deaths Rate Attributable to High Fasting Plasma Glucose by SDI
B
Deaths Rate Attributable to HBMI by SDI
C
Deaths Rate Attributable to Smoking by SDI
D
Deaths Rate Attributable to Secondhand Smoke by SDI
E
Deaths Rate Attributable to Low Physical Activity by SDI
F
Deaths Rate Attributable to Diet Low in Whole Grains by SDI
G
Deaths Rate Attributable to Diet Low in Nuts and Seeds by SDI
H
Deaths Rate Attributable to Diet Low in Fruits by SDI
I
Deaths Rate Attributable to Diet Low in Fiber by SDI
J
Deaths Rate Attributable to Diet High in Sugar-sweetened Beverages by SDI
K
Deaths Rate Attributable to Diet High in Red Meat by SDI
L
Deaths Rate Attributable to Diet High in Processed Meat by SDI
M
Deaths Rate Attributable to Alcohol Use by SDI
N
Deaths Rate Attributable to Low Temperature by SDI
O
Deaths Rate Attributable to High Temperature by SDI
P
Deaths Rate Attributable to Ambient Particulate Matter Pollution by SDI
Q
Deaths Rate Attributable to Household Air Pollution from Solid Fuels by SDI
Age-standardized Deaths Rate per 100k Population
SDI

Supplementary Figure 4. Death rate attributable to Level 4 risk factors of China by SDI. **(A)** Death rate attributable to high fasting plasma glucose (HFPG); **(B)** Death rate attributable to high body mass index (HBMI); **(C)** Death rate attributable to smoking; **(D)** Death rate attributable to secondhand smoke; **(E)** Death rate attributable to low physical activity; **(F)** Death rate attributable to a diet low in whole grains; **(G)** Death rate attributable to a diet low in nuts and seeds; **(H)** Death rate attributable to a diet low in fruits; **(I)** Death rate attributable to a diet low in fiber; **(J)** Death rate attributable to a diet high in sugar-sweetened beverages; **(K)** Death rate attributable to a diet high in red meat; **(L)** Death rate attributable to a diet high in processed meat; **(M)** Death rate attributable to alcohol use; **(N)** Death rate attributable to exposure to low temperature; **(O)** Death rate attributable to exposure to high temperature; **(P)** Death rate attributable to ambient particulate matter pollution; **(Q)** Death rate attributable to household air pollution from solid fuels.

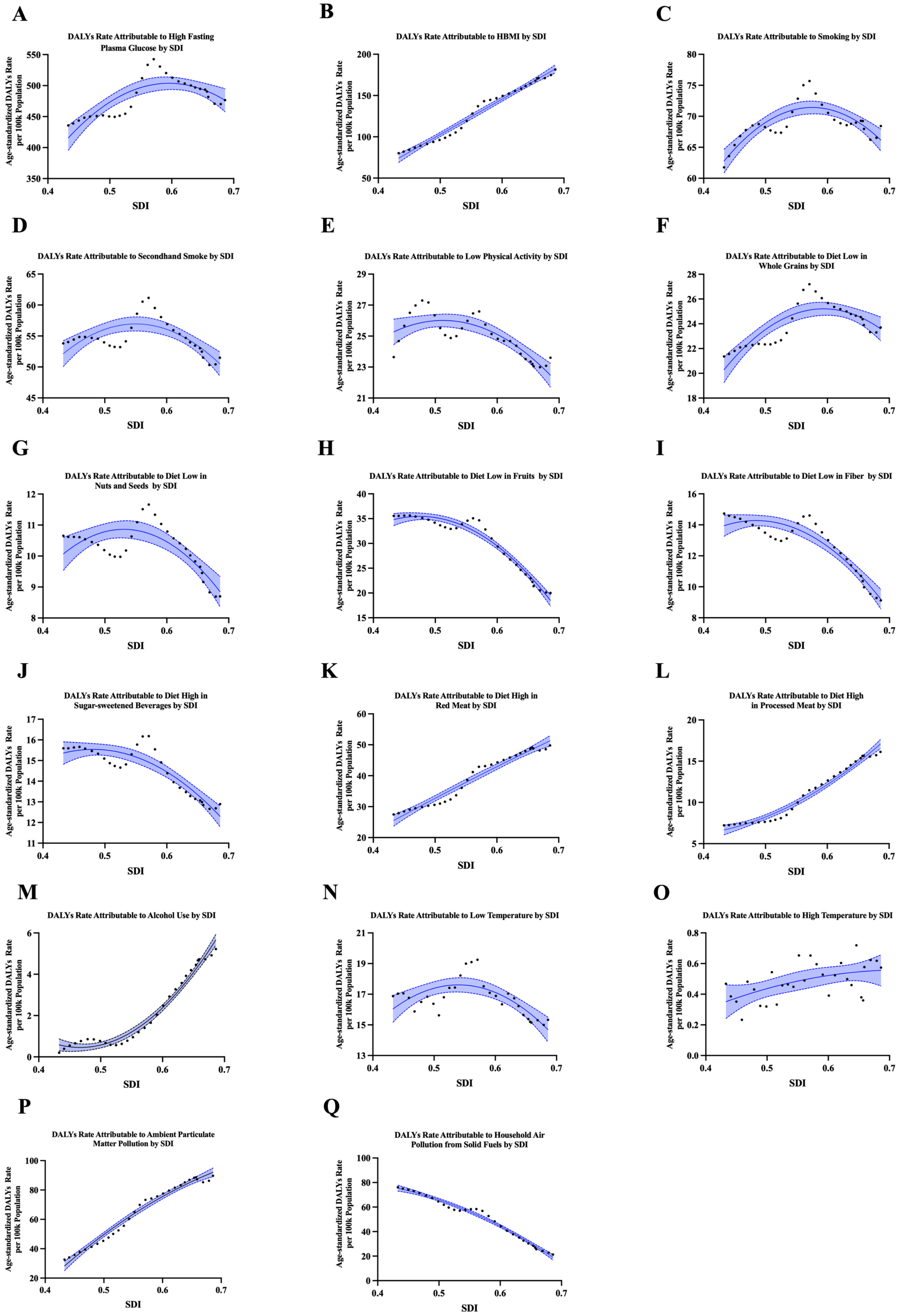

A
DALYs Rate Attributable to High Fasting Plasma Glucose by SDI
B
DALYs Rate Attributable to HBMI by SDI
C
DALYs Rate Attributable to Smoking by SDI
D
DALYs Rate Attributable to Secondhand Smoke by SDI
E
DALYs Rate Attributable to Low Physical Activity by SDI
F
DALYs Rate Attributable to Diet Low in Whole Grains by SDI
G
DALYs Rate Attributable to Diet Low in Nuts and Seeds by SDI
H
DALYs Rate Attributable to Diet Low in Fruits by SDI
I
DALYs Rate Attributable to Diet Low in Fiber by SDI
J
DALYs Rate Attributable to Diet High in Sugar-sweetened Beverages by SDI
K
DALYs Rate Attributable to Diet High in Red Meat by SDI
L
DALYs Rate Attributable to Diet High in Processed Meat by SDI
M
DALYs Rate Attributable to Alcohol Use by SDI
N
DALYs Rate Attributable to Low Temperature by SDI
O
DALYs Rate Attributable to High Temperature by SDI
P
DALYs Rate Attributable to Ambient Particulate Matter Pollution by SDI
Q
DALYs Rate Attributable to Household Air Pollution from Solid Fuels by SDI
Age-standardized DALYs Rate per 100k Population
SDI

Supplementary Figure 5. Disability-adjusted life-years (DALYs) rate attributable to Level 4 risk factors of China by SDI. **(A)** DALYs rate attributable to high fasting plasma glucose (HFPG); **(B)** DALYs rate attributable to high body mass index (HBMI); **(C)** DALYs rate attributable to smoking; **(D)** DALYs rate attributable to secondhand smoke; **(E)** DALYs rate attributable to low physical activity; **(F)** DALYs rate attributable to a diet low in whole grains; **(G)** DALYs rate attributable to a diet low in nuts and seeds; **(H)** DALYs rate attributable to a diet low in fruits; **(I)** DALYs rate attributable to a diet low in fiber; **(J)** DALYs rate attributable to a diet high in sugar-sweetened beverages; **(K)** DALYs rate attributable to a diet high in red meat; **(L)** DALYs rate attributable to a diet high in processed meat; **(M)** DALYs rate attributable to alcohol use; **(N)** DALYs rate attributable to low temperature; **(O)** DALYs rate attributable to high temperature; **(P)** DALYs rate attributable to ambient particulate matter pollution; **(Q)** DALYs rate attributable to household air pollution from solid fuels.
